# How Virtual Triage Can Improve Patient Experience and Satisfaction: A Narrative Review and Look Forward

**DOI:** 10.1089/tmr.2023.0037

**Published:** 2023-10-04

**Authors:** George A. Gellert, Joanna Rasławska-Socha, Natalia Marcjasz, Tim Price, Kacper Kuszczyński, Agata Młodawska, Aleksandra Jędruch, Piotr M. Orzechowski

**Affiliations:** ^1^ Infermedica, San Antonio, Texas, USA.; ^2^ Infermedica, Wroclaw, Poland.; ^3^ Infermedica, London, United Kingdom.; ^4^ Infermedica, Gliwice, Poland.

**Keywords:** patient satisfaction, virtual triage, symptom checker, patient experience

## Abstract

**Objective:**

To complete a review of the literature on patient experience and satisfaction as relates to the potential for virtual triage (VT) or symptom checkers to enhance and enable improvements in these important health care delivery objectives.

**Methods:**

Review and synthesis of the literature on patient experience and satisfaction as informed by emerging evidence, indicating potential for VT to favorably impact these clinical care objectives and outcomes.

**Results/Conclusions:**

VT enhances potential clinical effectiveness through early detection and referral, can reduce avoidable care delivery due to late clinical presentation, and can divert primary care needs to more clinically appropriate outpatient settings rather than high-acuity emergency departments. Delivery of earlier and faster, more acuity level-appropriate care, as well as patient avoidance of excess care acuity (and associated cost), offer promise as contributors to improved patient experience and satisfaction. The application of digital triage as a front door to health care delivery organizations offers care engagement that can help reduce patient need to visit a medical facility for low-acuity conditions more suitable for self-care, thus avoiding unpleasant queues and reducing microbiological and other patient risks associated with visits to medical facilities. VT also offers an opportunity for providers to make patient health care experiences more personalized.

## Introduction: The Rise of Health Care Consumerism

The rise of consumerism in health care has been accompanied by a wave of technology-enabled capabilities in the market, offering patients more choice, greater convenience, and opportunities for health care providers to offer a more streamlined consumer-patient experience. A negative patient experience in health care can yield a negative patient satisfaction or web review^1^—and a patient or family alienated from their care provider can increase risk of unnecessary and clinically inappropriate delays in diagnosis or treatment.^1^

Digital solutions could potentially have a positive impact on health care costs.^2^ On the other hand, as consumers increasingly share the health care cost burden, lack of a digital focus may have a negative impact on financial performance. Competitiveness in the health care market is increasing^3^ as patients play a more active, engaged role in their health care, seeking easier access and care navigation, greater convenience and service continuity, and better affordability. As a result, designing an effective, engaging, and positive experience for patient-consumers is in the interest of providers. Providers that choose to prioritize patient and e-health user experience will be better positioned to gain market share and increase the satisfaction of patients.

This review examines the literature with a focus on what has been most effective in improving health care and e-health experiences from the perspective of patients, and the potential role that virtual triage (VT) technology can play in delivering positive experiences that foster loyalty and bring value to the care delivery organization.

Health care delivery organizations are bringing more of their services online in the form of patient portals and digital front doors where VT is implemented to serve as the first point of contact for patients (or family members) with a health care delivery system. Individuals using a smartphone, tablet, or laptop to assess their symptoms on popular browsers are connecting with essentially random internet health care content of questionable scientific and clinical accuracy. However, a systematic VT engine is typically a multispecialty physician developed and certified digital tool that enables patient-users to more confidently and accurately secure a preliminary assessment of their symptoms, and which conveys reliable, actionable clinical care guidance.

## Background: The Emergence of Virtual Triage and Care Referral

VT is a digital technology that can be engaged by patients 24/7/365, and is accessible from any device with internet connectivity to assist in evaluating their symptoms and determining the appropriate level or acuity of care that is needed. Triage protocols and decision trees have been widely adopted by health care organizations as a guide for clinical decision making; however, advances in both consumer applications and artificial intelligence (AI) have enabled a new class of VT tools that are intended to be used directly by patient-users. While some attempts have been made to digitize traditional protocols and decision trees, this review will focus on AI-powered VT tools, which have been designed specifically for use by patient-users.

A VT classification algorithm considers the severity of the most likely conditions identified by an inference engine, as well as the occurrence of any particularly acute and alarming symptom or risk factor. The inference engine within VT software computes the probabilities of likely conditions, and automatically selects the most relevant questions to ask the patient-user next. At the heart of many VT inference engines is a statistical and probabilistic algorithm for advanced symptom assessment that uses the underlying knowledge stored in a medical knowledge base. The inference algorithm identifies which question to ask based on the patient's previous answers, demographics, medical history, and risk factors, using a value of information criterion. There is no prescribed VT interview or episode path and thus, in light of new evidence, the system can switch between various hypotheses (as human physicians do).

The primary function of a VT application is the conduct of a preliminary medical interview or triage query process, during which the patient-user answers questions regarding their sense of well-being and symptoms experienced. The answers provided are analyzed on a current basis, and within a few minutes, VT software conveys the analysis of symptoms and explains to the patient-user their probable causes, severity, and recommended level or acuity of medical care warranted. VT applications ask the patient-user to provide basic information, such as age, sex, risk factors, medical history, and symptoms.

On the basis of these responses, the preliminary medical interview or query process asks questions adjusted to the patient-user's specific case, focusing on the intensity of symptoms, their occurrence, and other related symptoms. The VT interview and episode conclude with a presentation of symptom analysis results and, in most VT engines, a recommendation for one of four levels of care, including self-care, consult a physician on an outpatient basis when next possible, proceed to an emergency department (ED), or call an ambulance for transport to an ED.

State of the art VT applications are fully scalable in terms of both the number of users and the volume of medical content. VT engines are classified under medical device class I in Europe, according to Medical Device Directive (93/42/EEC), and in the United States, under the Food, Drug & Cosmetic Act. The FDA currently exercises enforcement discretion, indicating that they are not required to comply with FDA regulations related to medical devices.

VT is superior to a generic internet browser search because patients are evaluated to identify and convey a triage level using an evidence-based set of clinical algorithms advising if—and how quickly—they should consult with a physician or other care provider, or if they could instead engage self-care (Table 1). In a few minutes, patients can verify their health status and care needs leveraging information and AI that has been extensively vetted and validated by physicians. VT enables health systems to provide patients with access to information and services beyond regular workday and daytime physician office working hours, or that involves potentially challenging travel to and possibly long waits in an ED. Research demonstrates that patients frequently utilize self-triage tools during weekends and evenings, and at night.^4^

**Table 1.** Comparison of Random Internet Medical Content Searches with Artificial Intelligence-Driven Virtual Triage

Characteristic	Common internet search engines	Evidence-based virtual triage
Input information	Single key words/phrases	Responses to highly specific clinical queries about multiple coexisting symptoms, pertinent medical history, and risk factors processed through algorithms engaging artificial intelligence
Information produced and conveyed to patient-users	Random information, unrelated to and uninformed by the user's clinical case and information	Evidence-based health assessment based on patient-user clinical, medical history, and demographic data
SEO	Based on the most popular searches and nonclinical SEO optimization	Independent of SEO, focused on and informed by individual user case and clinical information/symptoms and scientific evidence
Quality of clinical assessment and health care referral guidance	Difficult to assess, especially without medical knowledge and clinical understanding	Confirmed by specialty board-certified and licensed physicians, as part of a clearly defined and audited evidence-based quality management system
Time required to receive actionable information and clinical guidance	Varied, depending on the quality of results produced by search and the number of topics/issues searched	Several minutes (4–5), after which specific and clinically actionable recommendations are presented
Resulting health care seeking choices, behavior and actions	Unknown and not informed by a robust scientific evidence base and clinical expertise	Specific care referral to acuity appropriate and available clinical care services

SEO, search engine optimization.

VT can be especially helpful when individuals are uncertain if their symptoms warrant medical/physician or nurse attention, if and when their symptoms worsen, and when they are unsure of what to do. Patients may struggle to navigate the complexity of available medical services—which can be exacerbated given high variability in cost and health insurance coverage. A survey of users of online VT tools showed a reduction in uncertainty about the level and type of care needed from 34% to 21%.^5^

Commencing a patient's health care journey with VT can help guide patients about available and clinically suitable health care services. When well-integrated within the digital front door of a health care delivery system, VT can direct patients to very specific services and care settings available within the user's network, which have been informed by the patient's clinical presentation and needs. Research shows that patients tend to be overcautious in their health self-assessments, which may result in overuse of EDs.^6^ VT thus has the potential to either accelerate obtaining needed acute care or to reduce unnecessary health care seeking in settings of greater acuity, complexity, and cost than the individual's illness and chief complaint warrant from a clinical viewpoint.

While VT enables patients to more rapidly secure appropriate care, effective clinical triage can also potentially reduce patient volume pressure in high-demand service lines, such as the ED, improve the efficiency/appropriateness of clinical staffing, and possibly reduce clinician stress and dissatisfaction resulting when a large volume of patients present during already busy ED shifts who do not require emergent care. VT deployed strategically in health systems can also potentially help reduce patient leakage and maintain patients' engagement within a system network. Table 1 contrasts key differentiating features of general internet browser searches and VT in educating and guiding patient-users.

## Methods

This analysis approaches the topic of the potential of VT to improve patient experience and satisfaction as a narrative rather than a systematic review, because initial examination of the literature indicated that the volume of published peer-reviewed studies and journal literature—and particularly quantitative studies evaluating the impact of VT on patient experience/satisfaction—was slight and not substantive or extensive enough to warrant, or to enable, a systematic review of the literature. As this study involved only a review of existing published studies and no new patient data collection, IRB approval was not required and waived.

A thorough literature search was performed using the following keywords and related terms, including MeSH terms: *virtual triage*, *symptom checker*, *digital triage*, *patient satisfaction*, *patient experience*, *patient engagement*, *artificial intelligence*, *patient care*, *patient-centered care*, *patient safety*, *virtual care*, *digital health*, *healthcare*, *technology*, *empathy*, *virtual visits*, *health literacy*, *digital therapeutics*, *health care innovation*, and *physician-patient relationship.* The following databases were searched: Google Scholar, PubMed, Science Direct, ResearchGate, and Google. Literature was also identified by cross-referencing from key articles. Articles were included for review that focused on both the use of VT integrated within the patient engagement and intake process of a health system as well as standalone, nonaffiliated online VT services.

We included articles based on the following content criteria: (1) examination and discussion of important aspects of VT use contributing to level of patient satisfaction and experience; (2) examination and discussion of important aspects of patient satisfaction within clinical care (for example, clinician communication, clinician-patient communications, patient-physician relationship, quality of care delivery, health care personalization, patient-centered medicine, patient education, health care participation and coordination, and patient engagement); and (3) discussion of AI-driven health care solutions, including, but not limited to VT during patient care before, during, or after a care delivery episode.

For each article identified, the titles, abstracts, and keywords were screened by three co-authors for relevance. Articles selected for inclusion in the review were read in full text independently, and discussed and summarized by at least three co-authors. Articles were included in the final literature review and synthesis based on a consensus of reviewers, with final article inclusion or exclusion decided by the lead author. All co-authors contributed to the integration of included article findings into the review, as well as development and expression of the primary review theme of the potential value of VT in improving patient experience and satisfaction.

To create as comprehensive a picture of the discussed issues as possible, despite the limited literature on the subject, select nonpeer-reviewed publications and other online publications were included after a thorough vetting and verification of the credibility of cited sources.

Journal articles were sought and integrated into the review from 2011 onward. Of the 68 reference citations included, most were published from 2020 onward. Articles on VT that pre-date 2020, after which there have been major advances in the AI that drives VT, describe an obsolete technology far more rudimentary than the technology developed and reported on in the literature from 2020 onward. Thus, our primary focus was on 2020 and later published reports, which identified the potential for VT to positively impact patient experience and satisfaction. Earlier source articles were included to share background on the evolution of AI technology in medicine, and do not necessarily mention VT, but have enabled us to outline the background for the entire article.

Articles in languages other than English were excluded, as were those published before 2011. Other exclusion criteria included articles not covering VT and related technology solutions in the context of patient satisfaction.

## Results: Conceptualizing, Measuring and Valuing Patient Experience and Satisfaction

Defining patient experience involves consideration of a wide range of interactions that patients have with a health care system, including with physicians, nurses, other clinical and administrative hospital staff, physician practices, outpatient centers, ambulatory clinics, and other health care facilities. Patient experience engages an end-to-end journey, from symptom triage, patient intake to diagnosis, and treatment to outcome and care continuity. Health care organizations today face pressures to achieve positive clinical and financial results, which can lead to a focus on efficiency and financial performance overshadowing the patient experience. However, organizational and clinical research have demonstrated that patient experience influences clinical safety and quality, as well as financial performance.^7^

The impact of experience on health care access, care quality/safety, and cost requires a broader definition of health system user experience. The Beryl Institute segments patient experience into key domains.^8^ These include interactions, or the orchestrated touch points of people, processes, policies, communications, actions, and environment; and perception or what is recognized, understood, and remembered by patients and support staff, which varies based on individual experience, beliefs, values, and cultural background. Culture includes vision and values as expressed and manifest in the organization and the greater community. Finally, the continuum of care impacts patient experience before, during, and after the delivery of care.

VT may positively impact the patient's experience by lowering the need for in-person visits and thus potentially making health care more accessible and efficient,^9^ especially with VT-based self-diagnosis tools that require no staff involvement and are immediately available 24/7.^4^ This is comforting to patients—they are able to obtain information regarding their condition anywhere and anytime. Research shows that VT can serve as an effective tool for implementing patient-centered care and personalized medicine.^10,11^ In addition, tailored to the patient's needs, digital tools can be customized for users of diverse cultural or socioeconomic background. VT can also give the patient a sense of proactive engagement in the diagnostic and treatment process, which can have a positive impact on the patient's cooperation with medical staff and, ultimately, on treatment outcomes.^11^

### Measuring patient experience

Patient experience is measured through Consumer Assessment of Healthcare Providers and Systems (CAHPS) surveys, which are designed to assess patient experience with health care services delivered in different settings. The CAHPS database exists as a tool for providers to develop a better understanding of patient experiences in their specific setting, as well as a resource for consumers to make informed decisions when selecting providers and health plans.

In addition to surveys, there are other ways consumers express their satisfaction or dissatisfaction with their health care. Websites like Healthgrades, RateMDs, and Google My Business collect and publish reviews on health care providers. Consumers may perceive nonclinical ratings provided by commercial websites or social media as equally important as clinical ratings provided by government websites.^12^ Word-of-mouth recommendations also impact selection of a primary care provider, and a recent survey reported that 42% of respondents would rely on recommendations from their existing providers, while 29% stated that their top source of recommendations would be friends, family, and coworkers.^13^

While the terms “patient experience” and “patient satisfaction” are often used interchangeably—and are inextricably linked—they are not identical. Assessing patient experience requires a determination of whether something that should have occurred in care delivery (such as clear communication with providers) actually did and with what frequency. Satisfaction, however, concerns whether patient expectations about a health care encounter were met. Two individuals receiving the same care, but having varying expectations for how that care should be delivered may well convey different satisfaction ratings.

VT can help providers deliver a more human-centric patient experience by employing a customized engagement of the health system in early screening and detection, based on the specific symptoms and past medical history that the patient shares during the triage process. While VT is driven by standardized algorithms leveraging the clinical evidence-base, the AI it deploys conveys a more interactive, conversational, and personalized experience for patients, despite its automation. This can be observed in the increasing popularity of online VT or symptom checkers even before (but expanded by) the COVID-19 pandemic.

VT occurring at the outset of symptoms and perceived need of care can serve as a digital front door for patients. It enables providers to extend their engagement of patients early in the lifecycle of care delivery. VT typically evaluates reported patient symptoms and past medical history to arrive at several possible, ranked clinical diagnoses, and identifies the kind/level of care acuity that the patient-user should consider: self-care, outpatient care, or ED visit/ambulance. Some VT solutions use four triage categories, further dividing outpatient care into urgent (requiring medical care within 24 h) and nonurgent (recommending seeking care in the near future).^14^

VT can help reduce avoidable utilization in a care delivery setting that is not matched to clinical acuity need based on clinical presentation, particularly avoidable use of the ED as a vehicle for obtaining routine primary care. VT can also accelerate appropriate care delivery when high acuity or morbidity/mortality risk demands it. VT thus also enhances potential clinical effectiveness and can reduce avoidable care delivery due to late clinical presentation. Delivery of earlier and faster, more acuity level-appropriate care, as well as patient avoidance of excess care acuity (and associated cost), are positive contributors to patient experience and satisfaction.

### The value of improving patient experience

Increased understanding of the patient experience is essential to creating a patient-centered approach to health care. Importantly, patient experience correlates with improved clinical outcomes, medication adherence, and quality measures like readmissions.^15^ VT can potentially positively affect patient experience and satisfaction by making more efficient and effective use of time allocated for patient visits and reducing overall wait times.^16^

When employed in the initial evaluation of a patient, VT rapidly provides valuable information about the patient's health problems, disease risks, and major health complaints, enabling physicians to glean pertinent patient history and focus on the right problem. VT can potentially reduce time needed for a visit, increasing visit efficiency. Use of VT offers the opportunity to determine whether a patient's in-person visit is necessary in a given case, or if a virtual or telemedical encounter will suffice.^17^ This facilitates financial optimization by reducing costs in avoidable tests and radiological studies to confirm the initial diagnosis, as VT can potentially help guide the diagnostic and treatment process.^17^

When patients play a more active role in their health care, they tend to report a more positive experience, and patients who are more actively involved in their health care experience better outcomes.^18^ Studies of patients hospitalized for myocardial infarction found those with more positive care experiences had better health outcomes,^19^ and 43% of physicians reported well-informed patients had better health outcomes.^20^ VT should be an integral part of patient engagement to leverage the association of patient satisfaction with improved personal engagement.

Patient experience is also correlated with provider financial performance.^21^ Elevating patient experience can lead to increased commercial growth and revenue, with hospitals having high patient satisfaction scores achieving higher profitability. Hospitals with excellent CAHPS patient ratings between 2008 and 2014 had a mean net revenue margin of 4.7% compared to 1.8% for hospitals with low ratings.^22^ Patients tend to keep or change providers based on positive or negative experiences, and were three times more likely to leave a physician's practice based on negative experiences.^12,21^

Patients are more likely to write a negative rather than positive internet health care review.^23^ Positive patient experiences are associated with a lower risk of medical malpractice, with a 21.7% increase in hospitals being named in a medical malpractice suit when patient experiences were poor.^12,21^ Improved patient experience is also associated with greater employee satisfaction and reduced turnover, with a 4.7% reported reduction in employee turnover after focused efforts to improve patient experience.^12,21^ VT can enhance provider financial performance through increased patient satisfaction with improved pre-care and patient intake.

Research shows that the quality of communication during a medical visit affects the patient's experience and creates a positive perception of their relationship with the medical staff.^24^ VT accelerates the diagnostic process, leaving clinicians more time to talk to patients and explain their diagnosis and treatment plan. VT facilitates a more rapid narrowing of the patient's diagnosis, which can potentially affect the patient's view of provider services positively. It also delivers economic benefits by reducing unnecessary patient travel to medical facilities.^25^ When care is appropriately diverted from the ED or outpatient centers to self-care, the application of digital triage as a front door helps minimize patient need to visit a medical facility for low-acuity conditions, avoiding unpleasant queues and reducing communicable disease and other patient risks associated with visits to medical facilities.^25^

When VT is deployed within a health system, travel distance to and lack of clinical staff are removed as obstacles to patient engagement. Patients with limited physical mobility or transportation choices, who are elderly or hesitant to take time off from work to pursue care, can use VT to get expert medical advice from home or work. When VT is integrated with care management and related virtual health resources, providers benefit by enabling remote management of less severe, low-acuity conditions, reducing patient volume and throughput pressure at hospitals/EDs. Indeed, most physicians report that digital technologies improve patient care overall.^26^ Incorporating new digital technologies into clinical workflows has also eased the administrative burden on clinicians, allowing higher quality interaction with patients.

### The impact of consumerism on patient health care expectations

Patient consumerism increasingly shapes contemporary health care delivery. VT provides a more streamlined, convenient, personalized and navigable patient experience, and 75% of U.S. consumers wish their health care experiences were more personalized.^27,28^ The expanding use of VT is, in part, a response to growing patients' expectations regarding health care quality.^29^ In addition, there is growing awareness and familiarity with VT among patients as a convenient, easily accessible solution.^30^ With VT, patients receive real-time personalized feedback regarding their health condition and care/referral guidance that can help providers advance personalization of patient engagement.

VT enables providers to send individualized, personalized recommendations for the level of care that a patient-user requires based on the triage assessment, including self-care, outpatient office-based or clinic care, or urgent care either at an emergency department or by calling for an ambulance. Integrated with provider patient intake functionality, VT can remind patients of the need for preventive care or follow-up visits, and facilitate their scheduling, helping providers manage patients as health care consumers to deliver a more patient-centered and satisfying care experience.

Patient-consumers are not easily satisfied and increasingly unwilling to tolerate unnecessary bureaucracy and related inefficiencies.^27–29^ Providers who excel in supporting shared decision-making^31^ and who demonstrate they can empathize with the patient's point of view and respond to their issues will have competitive advantage in the era of patient centricity. VT enables efficient remote assessment of a patient's condition, and provides patient-users an opportunity to express their health problems without the public exposure, pressures, or rush that often accompanies other patient engagement modalities, including live conversations with traditional provider call centers. VT allows patient-users any amount of time to conduct a triage interview in privacy on an anytime basis.

As patients progress or escalate in level of care acuity needed, VT can expedite patient decision-making, accelerating delivery of appropriate care and lowering stress levels for patients and provider intake/registration staff. Linked with functionality automating completion and clerical filing of administrative forms and documents, VT can increase productivity while enhancing the quality of provider patient communications. As a technology, VT may potentially enhance empathy.^32^

## Key Contributors to Patient Satisfaction

### Health care accessibility

Accessibility is an important driver of patient satisfaction. During the pandemic, ease of accessibility was enhanced by expanded virtual and telemedical access, and may have increased ongoing patient expectations, including near real-time communication with their physicians. Online chat services and digital intake solutions to check in before appointments, provision of self-service options through virtual assistants, VT/symptom checkers, automated follow-up patient communications, medication adherence reminders, and other solutions have all substantially improved patient health care accessibility.

Patients with health insurance are 61% more likely to go directly to urgent care than those without insurance, while uninsured patients are 90% more likely to try self-diagnosis through internet searches.^33^ Accessibility can be increased by making digital tools much more readily available to patients from diverse socioeconomic backgrounds, with disabilities, facing health care inequities, with low health literacy levels, or for whom English is a foreign language.^34^

Providing alternative, clinically validated options for diagnosing symptoms and guiding care seeking can increase health care access and drive more appropriate health care use. VT may increase accessibility of health care, and helps patients avoid unnecessary in-person ED visits.^35^ Remote management of chronic conditions may be positively perceived by patients and improves outcomes.^36^ VT can help resolve problems in rural areas with limited/difficult access to health care, frequently eliminating the necessity to travel to a medical facility.^37^ VT reduces patient wait time for engagement and feedback on their health situation, and enables prioritization of care for more severe clinical presentations. By helping to define a patient's health needs, VT clarifies the need for specialist care and reduces waiting times for specialist consultation.

### Health care coordination

Patients often perceive their care as disjointed and fragmented, with nearly half of patients citing concerns about poor communication and having to explain their symptoms repeatedly to different provider staff members.^33^ Fragmentation of care is a systemic problem, and despite provider efforts to deliver a seamless experience, patients often need to navigate siloed one-off care solutions, rather than a common platform that connects disparate parts of their journey in a more integrated manner. VT creates an individual patient care profile, including disease risk factors and past medical history, enabling efficient communication to support better coordination and continuity of interdisciplinary care. VT can serve as an effective patient follow-up tool after treatment or hospitalization.^38^

### Health care personalization

Patients prefer personalized services that help them find the right solutions to their care needs, and VT can help personalize care and thereby improve patient experience.^39^ VT makes care navigation and delivery easier and more intuitive for patients. VT helps providers reach and serve patients early in the course of illness and before a potential in-person care visit, analyzing their symptoms, identifying possible conditions, and recommending appropriate care. This personalized, data-driven approach to care can help ensure patients get appropriate care they need. VT tools enable identification of the preventive and treatment needs of patients, providing them with individualized, personalized education to promote healthy lifestyles, including dietary and physical activity advice, as well as preventive testing or health promotion recommendations for specific risk groups.

### Health care participation

Patients seek to have a role and a level of control in the care services they receive, and to be involved in their care/treatment decisions.^18^ Involving patients in the care decision-making process improves the patient-clinician relationship and patient satisfaction, lowers medical costs 5.3% and reduces avoidable hospital admissions by 12.5%.^18^ Engaging patients in shared decision-making helps providers drive positive health outcomes. VT enables effective communication with patients about current health care needs, and promotes active participation in the treatment process. Sharing responsibility for care management decisions with patients promotes informed and proactive attitudes to care received.^40^

### Patient education

In the absence of effective education about their symptoms and illness, patients can feel confused about their care pathway and need for treatment, adding stress and frustration, which can contribute to a negative experience. Low health literacy impacts the degree to which patients have the capacity to obtain and understand health care information needed to make care decisions.^41^ One-third of the U.S. population has low health literacy, often patients with lower socioeconomic status and income, education, or English proficiency. A fourfold increase in health care costs, with 6% more hospital visits and an average 2-days longer stay in hospital have been reported among patients with low health literacy.^42^ Empowering patients with the knowledge and skills needed to feel (and be) better informed about their condition and treatment is important to provider efforts to increase the excellence and cost-effectiveness of care delivered.^42^

VT allows more precise patient profiling of treatment and prevention needs and health risks, and can help advance patient education on healthy lifestyles.^43^ Research suggests AI-driven tools like VT can be beneficial in managing patients with chronic diseases, leading to better health outcomes and cost savings.^43^ VT can provide patients personalized, reliable care guidance and preventive health information about regular health checkups, need for clinical follow-up, or vaccination. VT may also potentially decrease avoidable clinical exacerbations among at-risk patients. The use of digital tools, including VT, improves symptom monitoring, thus enabling more timely intervention.^44^ VT can help patients better understand and prioritize the urgency of needed care visits, thus helping patients avoid unnecessary care and potential complications (Table 2).

**Table 2.** Summary of Key Contributors to Improved Patient Experience and Satisfaction

Aspect of care	Key contributors to patient satisfaction	Virtual triage and care referral value add
Health care accessibility	Virtual care and telemedicine Online chats Digital intake Virtual triage and care referral Automated patient follow-up communications Self-service options	Can reduce unnecessary in-person visits, travel, and office wait times Improves patient need and care acuity matching Can be integrated with health system intake processes and technology Can help overcome inequities and other barriers to care
Health care coordination	Virtual follow-up Quality of communication; not having to explain the same information to successive members of the medical team	Enables remote patient monitoring May improve communication
Health care personalization	Care personalization	May serve as care personalization tool by making care navigation easier and more intuitive for patients May enable identification of preventive and treatment needs May promote healthy lifestyles in a personalized manner
Health care participation	Active engagement in the treatment process Involvement in the care decision-making processes; shared decision making Proactive engagement in the treatment process	Beneficial in managing patients with chronic diseases, leading to better health outcomes and cost savings
Patient education	Patient education on healthy lifestyles	Eases and improves management of patients with chronic diseases

## Discussion: Challenges and Opportunities in Deploying VT to Improve Patient Satisfaction

Implementing new virtual technologies requires time^45^ and collaboration with care delivery teams; otherwise, they may fail to have a favorable impact on patient satisfaction. A collaborative approach with continuous input from diverse provider stakeholders is critical to implementation success. Health care delivery teams should be familiarized with the benefits of using VT technology,^46^ including demonstrating the potential for streamlining workflows and care delivery, which impact patient satisfaction. Regulations governing data security and compliance have not been updated to include the vast amount of patient data collected by virtual health tools.^47^

The COVID-19 pandemic provided a tipping point for widespread adoption of virtual health, telehealth, and remote patient monitoring, with providers seeing 50–175 times the number of remote patients than pre-pandemic.^48^ Consumers welcomed this new accessibility and convenience, with 88% wishing to continue using telehealth for nonurgent consultations as COVID-19 receded.^49^ Patients have come to expect the same agility and ease in their health care experiences as provided in other industries such as retail, with 82% stating health care should be easily accessible on their mobile device, 77% wishing they could text message their physician, and 69% desiring virtual physician visits.^50^

Patients not only seek increased frequency of virtual health engagement but also improved quality, with 28% changing providers due to poor digital experiences, and 65% indicating they would be very likely to recommend their provider after a great digital experience.^51^ Younger generations are accustomed to a high-quality digital experience—and increasingly demand it.^52^ Convenience is paramount for many, and they will readily share their personal health data in return for high-quality service and immediate attention.

VT is immediately available 24/7 to any patient, reducing the frustration of waiting for telephonic contact with a provider. VT can consider several factors in an integrated manner, including the patient's current condition and symptom severity, location, and health insurance status can guide the patient to the right specialist, enable scheduling of a telehealth appointment, and recommend the optimal acuity level of care for a patient's health problem. These factors can contribute to positive patient experience and satisfaction.

The pandemic clearly demonstrated that a large proportion of care can be delivered live online, meeting people where they are, with more convenience and less patient effort, helping patients identify and connect with the resources they need. Leveraging technology such as VT to provide better patient experiences is no longer an optional luxury—it is imperative. Half of surveyed patients in one study responded that a bad digital experience with a provider ruined their entire experience, while a good digital experience improved satisfaction,^53^ and 92% in another study stated that improving their experience should be a priority when providers deploy digital health tools.^54^

As digital transformation initiatives accelerate, so do patient expectations of engaging with providers digitally. VT can enable patients to have the positive experiences they desire/need using a next-generation care management platform that engages patients and saves providers time by integrating patient data, facilitating communication, and increasing access to linked patient resources. Technologies such as VT can help providers improve patient experience using the following strategies:

### Leveraging real-world, actionable data

In health care settings, collecting patient data helps providers monitor their patients remotely and allows them to provide immediate feedback. Frequent patient data capture, for example through VT, supports and informs clinical decision-making, while also potentially reducing risk of medical errors. VT can convey a more complete picture of patient status and experience by collecting data at multiple points of interaction along the care continuum in both analog and digital environments, enabling providers to identify areas requiring improvement. Capturing patient data *per se* is no longer enough—it needs to be collected in a way that is easy, intuitive, secure, and actionable, and that conveys value to the patient. Data capture by VT should be an integral component of provider efforts to constructively leverage real-world actionable data to increase patient engagement, derived value, and satisfaction.

### Engaging patients earlier in the care journey

Providers have recognized the value of offering new ways for patients to engage with health systems beyond conventional channels, and this has led to increased investment in establishing digital front doors to their services. With 40% of patients reporting before VT that they did not know what acuity level of care to seek,^55^ VT creates greater patient access to care when and where they need it, encouraging patients to engage earlier in their care journey. VT tools that have undergone rigorous clinical evaluation and standardization may have significant potential to improve patient care pathways when integrated with patient support in health care decision-making.^56^

Digital front doors, including VT engines, have improved accessibility and allowed patients to communicate with care teams, schedule appointments, review laboratory and test results, pay care bills, and conduct virtual/telehealth visits. Solutions are coming to market which will convey care further upstream, allowing providers to engage patients when they first experience symptoms of illness. Earlier identification of health risks and exacerbations of existing chronic illnesses will facilitate faster care delivery, which can improve patient outcomes, reduce costs, and alleviate some of the burdens facing providers today, in turn improving patient experience/satisfaction.

VT immediately provides patients with an initial explanation of their ailments at an early stage along with care guidance, translating into faster diagnosis and earlier treatment of patients with urgent health problems, and conveying reassurance when symptoms do not indicate a high-acuity need for care. Patients experiencing an increasing number or severity of symptoms can get quick feedback through VT when additional problems arise—with recommendations for earlier or urgent care referral for definitive diagnosis and needed treatment, as clinically appropriate.

### Streamlining patient-clinician communication

Given the importance of communication in health care, solutions that facilitate better interactions between patient and provider can add considerable value, whereas legacy systems, siloed data, lack of care integration, and misdirected messages cause communication breakdown and associated patient dissatisfaction. With the right virtual health solutions, providers can streamline communication, automate and personalize key parts of the patient journey, perform timely check-ins and follow-ups, and configure communications to better meet patient preferences and literacy levels. VT enables providers to meet patients where they are and engage them in that reachable moment, streamlining their communications and thereby facilitating faster, potentially better care delivery and a more satisfying experience for both. VT defines patients' needs and facilitates individualized engagement and communication.

### Personalizing care delivery

The increasing availability of social, environmental, and behavioral determinants of health data and the application of machine learning to these datasets are having a major impact on how services can be personalized to the specific needs of individual patients. From personalized medication guidance and dietary considerations to daily wellness/health promotion, providers can now build a holistic picture of the patient and proactively recommend specific services and education that align with where they are in their health care journey. VT creates an opportunity for providers to accurately determine patients' health needs and to implement personalized, patient-centered medical care. This can provide individual patients with a highly tailored health improvement and prevention plan that includes management of risk factors, lifestyle changes, improvements in eating habits, and physical activity recommendations.

AI will increasingly have an impact on personalizing patient experience. Call center communication technology is now able to provide real-time data to agents on patient sentiment, dynamically adjusting their communication style/tone according to the patient's live reaction, increasing the likelihood that patients leave the encounter feeling satisfied. VT AI is also being used to analyze social determinants of health data to better prepare clinicians before a patient encounter and guide them by sharing recommendations based on specific patient challenges and preferences. Automating reminders to keep patients compliant with their care plan and medications also conveys a degree of personalization that can improve patients' engagement and care experience/satisfaction. VT streamlines call center workflows by facilitating decision-making and gathers real-time feedback from patients, which can improve call center staff communication and problem resolution skills, benefiting patient satisfaction.

### Optimizing care coordination

Suboptimal care coordination impacts many aspects of patient experience, can lead to poor care outcomes, and hinders provider efficiencies and revenue generation. Lack of coordinated care may contribute to higher preventable morbidity among patients, resulting in avoidable hospital admission/readmission and ED use, which are costly to patients, payers, and health systems.^57^ Primary care providers, in particular, function as managers/gatekeepers of complex health conditions, and need to efficiently and accurately gather health information to direct patients to the right health care services and specialists at the appropriate time. Digital technology allows providers to communicate seamlessly with patients and specialists, facilitating delivery of more appropriate and effective care, and can eliminate barriers and bottlenecks in care delivery by automating communication while removing redundancies in information exchange and improving patient flow.

VT allows better monitoring of patient condition and earlier implementation of care interventions, potentially reducing avoidable ambulance trips and long wait times and high volumes in EDs by accurately determining the urgency/acuity of cases. Poor or incomplete understanding among patients of the implications and care needed for their clinical symptoms, and the appropriate level of care to seek, contributes to utilization of clinically unnecessary or inappropriate medical services.^58^ VT can help ensure appropriate acuity level use of care, as well as preserve continuity of care through improved communication and insights gained by timely capture of detailed characteristics of the patient's condition, which in turn can favorably impact patient satisfaction.

### Aligning digital services with clinical practice

The pandemic merged digital health with everyday clinical practices and enabled providers to become comfortable with virtual technologies to meet care needs. Digital tools need to align with clinical workflows and be simple to understand and use. Payers and providers acquired insights into what worked in pandemic telehealth and adjusted their digital services accordingly. Digital health and virtual care solutions must integrate into existing organizational processes, revenue models, and clinical workflows. Routinely delivering a consistent patient experience across asynchronous service components is central to offering a satisfying care experience. Providers who are thoughtful in their service redesign, ensuring digital and analog services are well integrated to drive consistency in care experience, are positioning to achieve high patient satisfaction.

VT can also play an important role during emergent crises such as the COVID-19 pandemic (or other communicable disease outbreaks) by educating patients about how to minimize risk of infection,^59^ by providing specific recommendations for reducing their risk with existing health conditions, and by diverting unneeded low-acuity patient visits away from acute care facilities. Queues and crowds in waiting rooms are associated with higher infection risk and can be minimized.^16,60^ This is critical in patients with heightened susceptibility to infection, such as immunosuppressed or elderly patients.^16^

### Adding a digital back door

Digital transformation in care delivery also focuses on what happens following a health care encounter. Timely post-visit follow-up and patient education deliver a high-quality end-to-end experience, and can potentially drive patient adherence to treatment plans, reduce avoidable readmissions and improve outcomes. A new wave of virtual follow up care solutions offers seamless automated ways for patients and providers to stay connected after a visit, and are designed to help patients secure the care guidance and support they need to effectively adhere to their prescribed treatment plans.

As health care continues to migrate away from the hospital and clinic and into the home and workplace, technology in the form of virtual care services, remote patient monitoring tools, and digital engagement applications such as VT can help providers scale those services and deliver more personalized care wherever and whenever patient need exists. VT allows for continuity of medical care from reporting of first symptoms through diagnosis and treatment, to follow-up after treatment or hospitalization. VT enables rapid, clear communication of care recommendations, reminders for appointments, check-ups and medications, immunizations and other preventive care, as well as improved monitoring of at-risk patients.

### Deploying telemedicine/virtual care to improve patient satisfaction

Although the COVID-19 pandemic forced more widespread adoption of virtual care and telemedicine, this digital transformation was inevitable and continues to accelerate. While a majority of patients value telehealth options currently available, research suggests there remain myriad ways they could be improved to provide patients with a better care experience.^61^ A survey of 1.3 million patients found 89% were satisfied with their physician after a telehealth visit, and 76% would recommend a videocall appointment after attending one.^61^ Yet, despite increasing demand for telemedicine, it accounts for less than one-quarter of patient care, suggesting that while patients enjoy the convenience of telehealth, logistical snags such as scheduling and poor audio/video connection may impede broader adoption.

Strategies that providers can use to alleviate this include creating a central resource team to ensure proper staffing and workflow support for telemedicine and virtual health operations. Re-envisioning and reengineering clinical triage processes and introduction of VT can help by offering telehealth engagement as an option when acuity appropriate, and automating steps in a more seamless, streamlined experience.

For certain conditions such as upper respiratory infections, influenza, allergies, depression and anxiety, telehealth can have a substantial role, allowing the clinician to diagnose the patient and issue an e-prescription, if appropriate.^62^ Digital and VT solutions can help clinicians make informed decisions rapidly and divert patients to more clinically appropriate and convenient virtual or self-care options, rather than scheduling an in-person office appointment or visiting an ED. This conserves patient time and reduces possible costs, and enables more clinically and cost-effective use of finite clinician time, allowing clinicians to focus on higher acuity cases.

Preparation of the patient and clinician is critical to the success of telehealth implementation. For example, when staff completed a day-before trial run visit with first-time telehealth patients, the call completion rate approached 100% and visit completion increased from 60% to 96%.^63^ Digitizing the intake process also reduces different clinicians asking the same questions repeatedly, expedites care, and decreases patient irritation.

Adopting telemedicine requires reframing the way providers regard the care continuum, where a hybrid model of in-person and telehealth visits becomes commonplace. Among patients who regularly receive telemedical care, 80% reported a virtual visit experience being as satisfying as an in-person one.^64^ Effective user interface design is essential to ensure a satisfying patient-user experience. A robust care management platform that supports telehealth services is rapidly becoming an essential component in creating a seamless telehealth experience for patients. VT makes it possible to plan and implement hybrid health care,^37^ particularly beneficial among less mobile individuals such as disabled patients and the elderly.

### Creating patient centricity

While health systems struggle to implement patient-centric solutions, patients now have more care choices than ever, with major retailers such as Walmart, Consumer Value Stores (CVS), and Amazon offering consumer-centric options such as “one-stop shopping” care.^65^ Providers need to engage the kind of customer focus that these traditionally consumer-centric competitors offer. VT can include patient intake that streamlines and integrates patient data collection such that it can fit seamlessly into clinical workflows, benefiting both patients and providers. Equipped with intelligent algorithms, VT frees physicians from asking repetitive questions by providing essential patient data before consultation, minimizing administrative tasks during the visit, and freeing up more time to spend with patients.

Provider call centers have historically been a solution for managing administrative tasks such as appointment scheduling and reminders, very basic triage, and health care referrals, but have not optimized and streamlined care delivery. Call centers rely on costly clinical staff to provide 24/7 coverage. New technologies such as VT offer much advanced triage and referral of patient inquiries, directing patients to seek emergency care, in-person outpatient office or clinic care, self-care, or a telemedicine/virtual care visit. Thus, these technologies can reduce health care-related anxiety, as patients are guided correctly from the very beginning of their episode of perceived care need, which motivates them to seek and engage appropriate care.^66^

By automating patient triage and care referral through VT that is well integrated with call centers, administrative tasks such as receiving and responding to patient calls, emails, and other communications can be shifted away from the care delivery team, allowing providers more time to focus on the actual delivery of patient care. The VT process itself is faster and more efficient than many existing care pathways, increasing patient satisfaction and potentially identifying patients who need urgent care more rapidly, improving care effectiveness and safety.^67^ Robust call center systems with integrated VT can reduce costly avoidable ED visits, making health system operations more efficient and increasing patient satisfaction. Such implementations lead to an overall redistribution of traffic in health care, streamlining it and encouraging patients to seek appropriate care.^68^ VT can be a tool for streamlining call center operations by supporting complex decision-making processes.

## Conclusion

Clearly much can be gained for providers by increasing their focus on patient experience and satisfaction, which in turn impact clinical and market reputation, as well as financial performance. By embracing digital transformation and implementing solutions like VT that focus centrally on patient experience, providers can address key barriers to the delivery of high-quality, patient-satisfying care. Adoption of digital intake, AI-driven and automated VT, and care referral are digital health tools that can be leveraged by providers to create a more streamlined and satisfying experience for patients across the continuum of care.

## Authorship Contribution Statement

All co-authors collaboratively researched and wrote the article.

## Abbreviations Used

AI: artificial intelligence
CAHPS: Consumer Assessment of Healthcare Providers and Systems
ED: emergency department
VT: virtual triage
